# Incidence and risk factors of symptomatic knee osteoarthritis among the Chinese population: analysis from a nationwide longitudinal study

**DOI:** 10.1186/s12889-020-09611-7

**Published:** 2020-10-01

**Authors:** Yan Ren, Jiang Hu, Jing Tan, Xiaoming Tang, Qianrui Li, Huazhen Yang, Chunrong Liu, Qiao He, Kang Zou, Xin Sun, Bo Tan

**Affiliations:** 1 Chinese Evidence-based Medicine Center and National Clinical Research Center for Geriatrics, West China Hospital, Sichuan University, Chengdu, 610044 Sichuan China; 2 Department of Orthopedics, Sichuan Provincial People’s Hospital, University of Electronic Science and Technology of China, Chengdu, 611731 Sichuan China; 3 Department of Nuclear Medicine, West China Hospital, Sichuan University, Chengdu, Sichuan China; 4 West China School of Public Health, Sichuan University, Chengdu, 610041 China

**Keywords:** Symptomatic knee OA, Incidence, Chinese population, Risk factor

## Abstract

**Background:**

Knee osteoarthritis (OA) is a common disease condition associated with aging and a frequent cause of primary care consultations. Few longitudinal studies have been conducted to investigate the incidence of symptomatic knee osteoarthritis (OA) and to identify its risk factors among the Chinese population.

**Methods:**

The China Health and Retirement Longitudinal Study (CHARLS) is a nationwide longitudinal survey of persons aged ≥45 years. Symptomatic knee OA was diagnosed when both self-reported knee pain and self-reported physician-diagnosis arthritis existed. Using the national survey data collected from the CHARLS, we estimated the incidence of symptomatic knee OA, taking into account the complex survey design and response rate. We applied weighted logistic regression analysis to identify its risk factors.

**Results:**

In the 4-year follow-up, the cumulative incidence of symptomatic knee OA among middle-aged and older Chinese adults was 8.5%; the incidence was higher among females (11.2%) than males (5.6%). Female (odds ratio (OR) 1.98 [95% confidence interval (CI) 1.65–2.37]), rural area (OR 1.32 [95% CI 1.08–1.60]), and West region (OR 2.33 [95% CI 1.89–2.87]) were associated with a higher risk of incident symptomatic knee OA. Physical activities (OR 0.47 [95% CI 0.29–0.76]) and high education level (OR 0.60 [95% CI 0.41–0.88]) was associated with a lower risk of incident symptomatic knee OA, while histories of heart disease (OR 1.40 [95% CI 1.07–1.82]), kidney disease (OR 1.80 [95% CI 1.35–2.39]), and digestive disease (OR 1.54 [95% CI 1.30–1.82]) were associated with a higher risk of incident symptomatic knee OA.

**Conclusion:**

The cumulative incidence of symptomatic knee OA over 4 years was relatively high, and varied by province and region. Lack of physical activities was confirmed to be risk factors of incident symptomatic knee OA. The presence of heart disease, kidney disease, and digestive disease may be associated with a higher risk of incident symptomatic knee OA, further research need to confirm these findings.

## Background

China has the world’s largest elderly population, and the whole nation is aging fast – the proportion of aging population (≥60 years) was estimated to be 14.9% in 2013, 25.3% in 2030, and over 30% in 2050 [1]. Rapid aging has led to an increasing healthcare burden attributable to age-related diseases [2]. Knee osteoarthritis (OA) is a common disease condition associated with old age and is one of the leading causes of disabilities, which primarily affect elderly adults [3, 4]. The disease can substantially reduce quality of life [5]; severe cases may even lead to knee-joint replacement. The costs for knee OA management are usually very high, placing a heavy burden upon families and society [6–8].

Epidemiological studies to understand the burden of knee OA are currently of great importance for health-care policy makers and clinicians. A previous study using a nationwide survey found that the prevalence of symptomatic knee OA among Chinese adults aged ≥45 years was 8.1% and increased with age [9], and concluded that future studies are needed to identify the risk factors for incident symptomatic knee OA. To date, however, no data are available on the incidence of symptomatic OA among the middle-aged and older Chinese population. Additionally, due to significant imbalances in socioeconomic development, environmental conditions, lifestyle patterns, and health-care utilization among different geographic regions in China, large variations in the incidence of symptomatic knee OA may be present among these populations. The lack of information may hinder the effective planning and execution of health-care strategies, as well as efficient use of health care sources.

In addition, understanding the risk factors for knee OA is important for managing health among adults, particularly for elderly populations. Several longitudinal studies conducted in the U.S.A., the U.K., Japan, and Europe have investigated risk factors for incident symptomatic knee OA [10–13]. However, these studies were conducted in developed countries. The findings from them have limited implications for the Chinese population, because socioeconomic status, environmental factors, and lifestyle patterns differ substantially between the developing countries and the developed world. Although a few cross-sectional studies investigated factors associated with knee OA [9, 14–17], to date, no longitudinal studies have been conducted to examine risk factors for knee OA among the elderly Chinese population.

In order to bridge this important evidence gap, we conducted a cohort study analysis, using data from the China Health and Retirement Longitudinal Study (CHARLS), to investigate risk factors for incident symptomatic knee OA in Chinese adults aged 45 years or older. In addition, we determined the incidence of symptomatic knee OA for this population.

## Methods

### Data sources

The data sources of our study came from the CHARLS, a nationwide longitudinal survey among Chinese adults aged ≥45 years. A detailed description of the methodology was reported previously [18]. In brief, the CHARLS study employed a four-stage probability sampling approach to select representative samples of eligible participants. Specifically, the first stage involved a random sampling, using the probability-proportional-to-size (PPS) method, in all county-level units of China with the exception of Tibet, and a total of 150 counties were selected eventually. The sample was stratified by region and within region by urban or rural status and gross domestic product (GDP) per capita. The second stage randomly selected administrative villages in rural areas and neighborhoods in urban areas, as primary sampling units (PSUs), for which three PSUs were selected from each county. In the third stage, a random sample of 24 households was selected on the basis of geographic locations and lists of each PSU. Finally, a resident aged ≥45 years was randomly selected from a household, and an interview was undertaken with the selected resident and their spouse. Taking into account of the complex survey design and the non-response rate for the CHARLS, the weighted value was constructed from the sampling probability and response probability, and was provided by the CHARLS database.

In the CHARLS, the baseline survey was conducted in 2011, and 17,708 respondents were interviewed from 150 representative counties of 28 provinces across China. Using structured questionnaires, data were collected regarding demographic information, health status (e.g. self-reported general health, doctor-diagnosed chronic and infectious disease, lifestyle and life behavior, including sleep and physical activity), socioeconomic status and biomedical measurements (e.g. blood pressure, pulse, peak expiratory flow, height, weight, waist size). The respondents were followed up every 2 years through a face-to-face interview.

The current study is a secondary analysis of the CHARLS public data. All data collected in the CHARLS are maintained at the National School of Development of Peking University, Beijing, China. The datasets are available from http://charls.pku.edu.cn/pages/data/111/zh-cn.html. The CHARLS was approved by the Ethical Review Committee of Peking University, and all participants signed informed consent at the time of participation. No separate ethical approval was required for our study.

### Study population and outcome measurement

In our study, the outcome of interest was a reported symptomatic knee OA. We included all the participants in this study who were free from symptomatic knee OA at the baseline survey. The outcome symptomatic knee OA was ascertained if a participant responded with “yes” to the first two of the following questions, and responded “the knees” to the third question. (1) *Have you been diagnosed with arthritis or rheumatism by a doctor? (2) Are you often troubled with any body pain*? If the participant responded “yes” to the second question, they were presented with question (3): *On what part of your body do you feel pain? (list all body pains)* (Supplementary Table 1).

### Covariates

The data for our study included demographic information (gender, age, area of residence, and region), socioeconomic status (education), health status (underlying diseases, has undertaken some physical activities), and anthropometric measurements (height and weight). All the information was collected from reporting by the participants.

The covariates included gender, age, area, region of residence within the country, education, body mass index (BMI), having undertaken physical activities in the last month, and history of hypertension, dyslipidemia, diabetes, chronic lung disease, liver disease, heart disease, stroke, kidney disease, digestive disease, psychiatric disease, or asthma. The body mass index (BMI) was calculated as the individual’s weight divided by the square of their height (kg/m^2^). A person doing physical activities (e.g. dancing, body building) or not in the last month was categorized as “yes” or “no”. Self-reported history of health conditions at the baseline survey was categorized as “yes” or “no”.

### Statistical analysis

We categorized the following variables: age (<50 years, 50–59 years, 60–69 years, and ≥ 70 years), area (urban vs. rural), region of residence within the country (East vs. Central vs. West), education (no formal education vs. elementary school vs. middle school vs. high school or higher), and BMI by World Health Organization (WHO) criteria (underweight, normal, and overweight: < 18.5, 18.5–24.9, and ≥ 25.0 kg/m^2^). Because of a limited number in the obesity group (BMI ≥ 30.0 kg/m^2^), we combined obese and overweight participants.

Taking into account the complex survey design and the non-response rate for the CHARLS survey, we used the inverse probability weighting method (the Proc Surveyfreq procedure in SAS version 9.4) to calculate the weighted cumulative incidence of symptomatic knee OA and the weighted percentage of symptomatic knee OA. Then we used the Taylor linearized method to estimate the variance of weighted cumulative incidence. According to its variance, we calculated its 95% confidence interval (CI).

We used the Proc Surveylogistic procedure in SAS version 9.4 to examine the risk factors of incident symptomatic knee OA. The basic model can be written as follows:

Model1 $$ \mathrm{logit}\left(\uppi \right)=\log \left(\frac{\uppi}{1-\uppi}\right)={\beta}_0+\sum {\beta}_i{x}_i $$

Model2 $$ \mathrm{logit}\left(\uppi \right)=\log \left(\frac{\uppi}{1-\uppi}\right)={\beta}_0+\sum {\beta}_i{x}_i+\sum {\beta}_j{diseases}_j $$

The Surveylogistic procedure fits linear logistic regression models for discrete response survey data by the method of maximum-likelihood method. For statistical inferences, Proc Surveylogistic incorporates complex survey sample designs, including designs with stratification, clustering, and unequal weighting. In model 1, we validated some known risk factors, which have been reported in other studies, including gender, age, area, region, education, BMI group, doing physical activities in the last month. In model 2, we explored additional potential risk factors, which may be associate with the systematic knee OA through medication use, chronic inflammation, or other reasons, including histories of hypertension, dyslipidemia, diabetes, chronic lung disease, liver disease, heart disease, stroke, kidney disease, digestive disease, psychiatric disease, and asthma. Odds ratios (ORs) and 95% CIs were presented for variables in the models. We used a complete case analysis for the primary risk factors analysis.

We conducted two sensitivity analyses to confirm our results. With respect to missing data, we performed multiple imputation for those with missing items, under the assumption that data were missing at random. In order to reduce sampling variability from the imputation simulation, missing values were replaced by imputed ones from ten duplicate datasets. Then we compared the differences of missing data in demographic and clinical variables between the case and control group; we performed sensitivity analyses to estimate risk factors of incident symptomatic knee OA. With respect to respondents lost to follow up, firstly we assessed baseline characteristics between respondents lost to follow-up and respondents included in the final analysis; then, we performed sensitivity analyses for risk factor estimation.

## Results

**Fig. 1:**
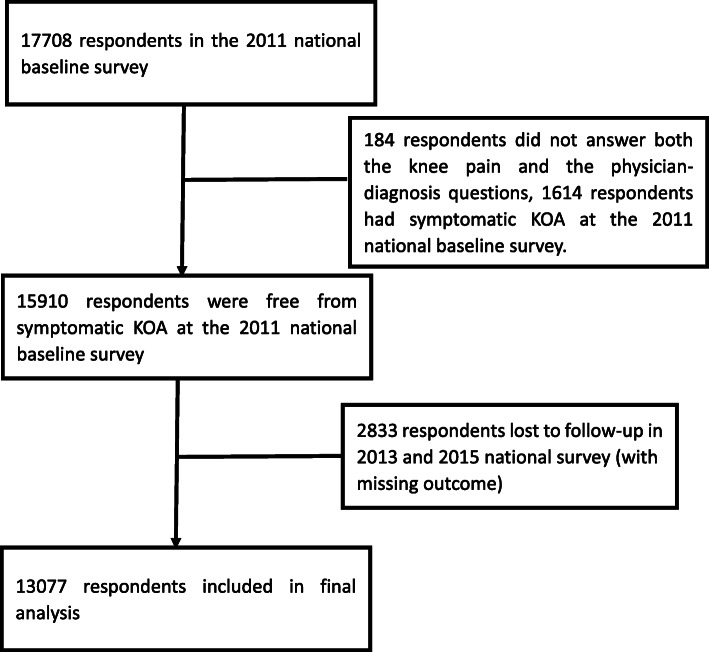
Flowchart showing the selection of the respondents who were included in the final analysis in this study

In the CHARLS, 15,910 respondents were free from the symptomatic knee OA at the 2011 national baseline survey, among whom 2833 were lost to follow-up in 2013 and 2015. As a result, 13,077 remained in the cohort until 2015 and were finally used in our study (Fig. 1).

Among the 13,077 respondents included in this analysis (Table 1): 6726 (51.5%) were women; the mean age was 64.2 years (SE 0.09); 8140 (62.3%) lived in rural areas. The respondents were generally equally distributed across geographic regions (36.4, 32.4, and 31.2% from East, Central, and West of China, respectively). Among all respondents, 40.0% completed elementary school only, and 12.0% received high school or higher education; 62.9% had normal weight (BMI: 18.5–24.9 kg/m^2^), 6.4% were underweight (BMI: < 18.5 kg/m^2^), and 30.7% were overweight (BMI: ≥25.0 kg/m^2^). Most respondents did not have pre-specified disease conditions at the baseline survey, as per physician diagnosis.

**Table 1:** Characteristics of respondents by status of symptomatic knee OA

Variables	All respondents			Symptomatic knee OA			Without symptomatic knee OA		
	No. (*n* = 13,077)	% unweighted	% weighted	No. (*n* = 1196)	% unweighted	% weighted	No. (n = 11,881)	% unweighted	% weighted
**Gender**									
Male	6347	48.55	48.15	396	33.11	31.71	5951	50.11	50.11
Female	6726	51.45	51.85	800	66.89	68.29	5926	49.89	49.89
**Age, years** (mean ± SE)	13,077	64.24 ± 0.09	64.45 ± 0.13	1196	64.92 ± 0.27	64.75 ± 0.32	11,881	64.17 ± 0.09	64.42 ± 0.14
< 50	908	6.94	7.33	83	6.94	6.81	825	6.94	7.38
50–59	4043	30.92	31.36	318	26.59	26.73	3725	31.35	31.79
60–69	4776	36.52	34.88	461	38.55	38.89	4315	36.32	34.51
≥ 70	3350	25.62	26.43	334	27.93	27.57	3016	25.39	26.32
**Area**									
Urban	8140	62.25	54.68	396	33.11	67.11	5951	50.11	61.42
Rural	4937	37.75	45.32	800	66.89	32.89	5926	49.89	38.58
**Region**									
Central	4237	32.40	30.36	369	30.85	29.14	3868	32.56	30.47
East	4760	36.40	39.94	270	22.58	24.53	4490	37.79	41.37
West	4080	31.20	29.70	557	46.57	46.33	3523	29.65	28.16
**Education**									
No formal education	3441	26.31	25.23	430	35.95	34.96	3011	25.34	24.32
Elementary school	5230	39.99	39.31	529	44.23	45.14	4701	39.57	38.78
Middle school	2839	21.71	21.81	163	13.63	13.72	2676	22.52	22.56
High school or Vocational school or higher	1567	11.98	13.65	74	6.19	6.18	1493	12.57	14.34
**BMI group (kg/m**^**2**^**)**									
< 18.5	678	6.45	6.30	72	7.32	6.82	606	6.36	6.25
18.5–24.9	6615	62.90	62.65	609	61.95	61.38	6006	63.00	62.78
≥ 25.0	3223	30.65	31.04	302	30.72	31.80	2921	30.64	30.97
**Done some activities** (such as played a sport, social, or other kind of club dancing, doing physical exercise, doing Qigong,et al.)									
Yes	705	5.39	7.03	800	66.89	2.89	5926	49.89	5.66
No	12,372	94.61	92.97	396	33.11	97.11	5951	50.11	94.34
**Hypertension**									
Yes	2930	22.52	23.26	800	66.89	27.17	5926	49.89	22.15
No	10,079	77.48	76.74	396	33.11	72.83	5951	50.11	77.85
**Dyslipidemia**									
Yes	1129	8.80	9.31	800	66.89	10.30	5926	49.89	8.74
No	11,704	91.20	90.69	396	33.11	89.70	5951	50.11	91.26
**Diabetes**									
Yes	670	5.17	5.51	800	66.89	7.06	5926	49.89	5.15
No	12,291	94.83	94.49	396	33.11	92.94	5951	50.11	94.85
**Chronic lung**									
Yes	1130	8.67	8.66	800	66.89	12.80	5926	49.89	8.29
No	11,901	91.33	91.34	396	33.11	87.20	5951	50.11	91.71
**Liver disease**									
Yes	434	3.34	3.39	800	66.89	4.74	5926	49.89	3.15
No	12,559	96.66	96.61	396	33.11	95.26	5951	50.11	96.85
**Heart disease**									
Yes	1335	10.27	10.10	800	66.89	16.81	5926	49.89	9.65
No	11,668	89.73	89.90	396	33.11	83.19	5951	50.11	90.35
**Stroke**									
Yes	237	1.82	1.99	800	66.89	2.85	5926	49.89	1.73
No	12,811	98.18	98.01	396	33.11	97.15	5951	50.11	98.27
**Kidney disease**									
Yes	671	5.16	5.29	800	66.89	8.88	5926	49.89	4.75
No	12,334	94.84	94.71	396	33.11	91.12	5951	50.11	95.25
**Digestive disease**									
Yes	2671	20.48	19.88	800	66.89	30.48	5926	49.89	19.29
No	10,374	79.52	80.12	396	33.11	69.52	5951	50.11	80.71
**Psychiatric disease**									
Yes	166	1.27	1.53	800	66.89	1.62	5926	49.89	1.22
No	12,860	98.73	98.47	396	33.11	98.38	5951	50.11	98.78
**Asthma**									
Yes	394	3.02	3.01	800	66.89	5.01	5926	49.89	2.86
No	12,636	96.98	96.99	396	33.11	94.99	5951	50.11	97.14

### Cumulative incidence of symptomatic knee OA over 4 years

In the 4 years of follow-up, 8.5% (7.7–9.3%) of participants developed symptomatic knee OA (Table 2). The cumulative incidence over 4 years was higher among females (11.2%) than males (5.6%). Respondents aged 60–69 years had the highest incidence, with 9.5% being affected with symptomatic knee OA. Respondents who resided in rural areas (10.4%) had a greater cumulative incidence of symptomatic knee OA than those in urban areas (6.2%). Those from the West (13.2%) and Central regions (8.1%) had a greater cumulative incidence of symptomatic knee OA than those from the East region (5.2%). Cumulative incidence was much lower among respondents who had received a longer duration of education or undertook physical activities (e.g. dancing). The respondents who were affected by a physician-diagnosed disease of other diseases at baseline survey had a greater cumulative incidence.

**Table 2:** The cumulative incidence over 4 years of symptomatic knee OA by age, area, region, education, BMI group, activities and chronic disease (Values presented are the weighted incidence of symptomatic knee OA)

	Incidence (%) (95%CI)		
	Women	Men	Total
**Total**	11.16 (10.15–12.26)	5.58 (4.87–6.39)	8.47 (7.74–9.26)
**Age, years**			
< 50	10.17 (7.55–13.57)	4.08 (2.46–6.69)	7.87 (5.95–10.35)
50–59	9.25 (7.79–10.94)	4.77 (3.73–6.08)	7.22 (6.19–8.42)
60–69	12.88 (11.12–14.88)	6.10 (5.10–7.28)	9.45 (8.36–10.65)
≥ 70	11.77 (10.08–13.71)	6.07 (4.93–7.45)	8.84 (7.75–10.07)
**Area**			
Urban	8.55 (7.25–10.07)	3.51 (2.71–4.53)	6.15 (5.26–7.17)
Rural	13.36 (11.94–14.92)	7.27 (6.27–8.42)	10.40 (9.40–11.50)
**Region**			
East	7.08 (5.66–8.82)	3.21 (2.41–4.26)	5.20 (4.27–6.33)
Central	10.75 (9.32–12.38)	5.30 (4.30–6.51)	8.13 (7.19–9.19)
West	17.01 (15.08–19.13)	9.1 (7.57–10.9)	13.22 (11.82–14.75)
**Education**			
No formal education	12.42 (10.82–14.23)	7.40 (6.29–8.68)	9.73 (8.73–10.83)
Elementary school	12.67 (11.09–14.43)	8.23 (6.29–10.71)	11.74 (10.39–13.25)
Middle school	7.51 (5.91–9.49)	3.83 (2.97–4.93)	5.33 (4.46–6.36)
High school or Vocational school or higher	6.82 (4.72–9.75)	2.25 (1.46–3.45)	3.84 (2.85–5.15)
**BMI group (kg/m**^**2**^**)**			
< 18.5	9.63 (6.75–13.55)	9.43 (6.44–13.59)	9.53 (7.29–12.38)
18.5–24.9	10.77 (9.52–12.16)	6.54 (5.62–7.59)	8.62 (7.77–9.56)
≥ 25.0	12.35 (10.35–14.66)	4.23 (3.20–5.57)	9.02 (7.69–10.55)
**Done some activities** (such as played a sport, social, or other kind of club dancing, doing physical exercise, doing Qigong,et al.)			
Yes	5.74 (3.59–9.05)	0.95 (0.38–2.38)	3.48 (2.27–5.31)
No	11.58 (10.53–12.72)	5.92 (5.19–6.76)	8.85 (8.10–9.66)
**Hypertension**			
Yes	13.72 (11.45–16.35)	5.64 (4.41–7.19)	9.85 (8.46–11.44)
No	10.36 (9.3–11.51)	5.48 (4.72–6.36)	8.00 (7.25–8.83)
**Dyslipidemia**			
Yes	14.79 (10.46–20.5)	3.57 (2.37–5.35)	9.28 (6.92–12.34)
No	10.76 (9.72–11.9)	5.62 (4.89–6.45)	8.29 (7.54–9.10)
**Diabetes**			
Yes	16.37 (10.16–25.31)	4.37 (2.57–7.34)	10.83 (7.33–15.73)
No	10.87 (9.84–11.99)	5.59 (4.87–6.42)	8.31 (7.57–9.12)
**Chronic lung disease**			
Yes	17.61 (14.13–21.74)	9.00 (6.70–11.98)	12.51 (10.40–14.98)
No	10.69 (9.68–11.81)	5.15 (4.47–5.94)	8.08 (7.36–8.87)
**Liver disease**			
Yes	15.31 (10.59–21.62)	9.09 (6.06–13.43)	11.78 (8.74–15.69)
No	11.04 (10.01–12.16)	5.36 (4.67–6.16)	8.32 (7.59–9.12)
**Heart disease**			
Yes	19.03 (16.12–22.32)	6.94 (4.83–9.86)	14.02 (12.00–16.32)
No	10.14 (9.13–11.24)	5.35 (4.63–6.17)	7.79 (7.07–8.59)
**Stroke**			
Yes	13.09 (7.54–21.76)	11.19 (6.42–18.80)	12.11 (8.16–17.61)
No	11.14 (10.12–12.24)	5.47 (4.76–6.29)	8.41 (7.69–9.20)
**Kidney disease**			
Yes	18.52 (13.48–24.9)	10.82 (7.75–14.93)	14.19 (11.33–17.64)
No	10.81 (9.80–11.91)	5.21 (4.51–6.00)	8.13 (7.42–8.91)
**Digestive disease**			
Yes	16.98 (14.85–19.34)	8.2 (6.62–10.12)	12.95 (11.44–14.63)
No	9.63 (8.58–10.80)	4.92 (4.18–5.77)	7.33 (6.61–8.13)
**Psychiatric disease**			
Yes	11.74 (5.9–22.00)	1.71 (0.22–12.09)	8.99 (4.93–15.82)
No	11.16 (10.15–12.27)	5.55 (4.85–6.34)	8.44 (7.71–9.22)
**Asthma**			
Yes	21.05 (14.56–29.43)	9.29 (5.56–15.12)	14.10 (10.54–18.61)
No	10.93 (9.91–12.04)	5.44 (4.73–6.25)	8.30 (7.57–9.09)

**Fig. 2:**
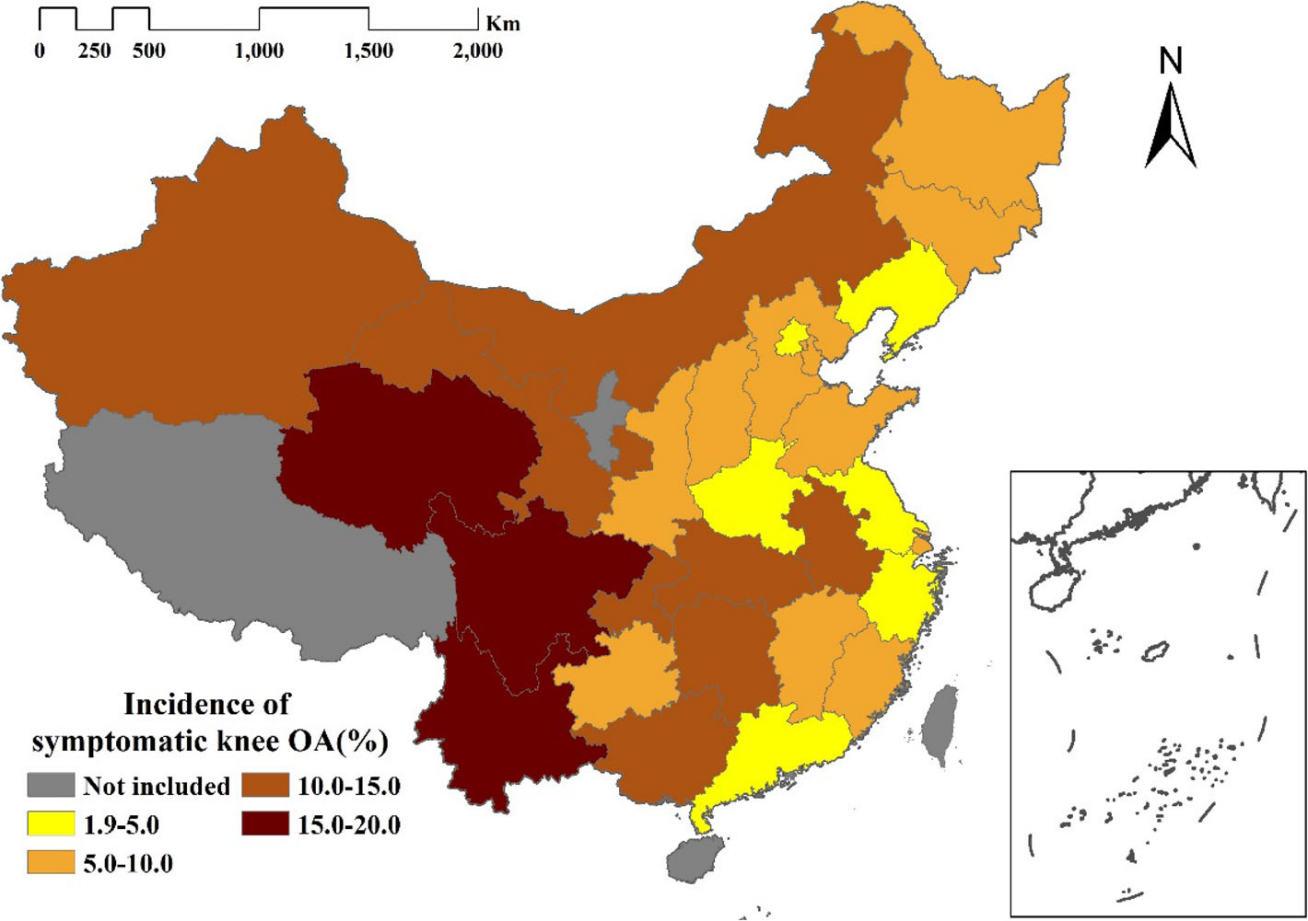
The cumulative incidence of symptomatic knee OA in different provinces of China. Notes. The density maps were generated by DataMap for ArcGis 10.1, using the Chinese geographic map template. An appropriate license from ArcGis has been obtained

The cumulative incidence of symptomatic knee OA by province is presented in Fig. 2. The six provinces with the lowest cumulative incidences (< 5%) were Beijing, Henan, Jiangsu, Liaoning, Guangdong, and Zhejiang. The three provinces with the highest cumulative incidences (> 15%) were Sichuan, Qinghai, and Yunnan.

### Identification of risk factors

The multivariable weighted analyses showed that gender, area, region, education, not having undertaken any physical activities, and self-reported histories of heart disease, kidney disease, and digestive disease were significantly associated with incident symptomatic knee OA (Table 3). The risk of incident symptomatic knee OA was higher in women (OR 1.98, 95% CI 1.65–2.37), rural areas (OR 1.32 [95% CI 1.08–1.60]), West (OR 2.33 [95% CI 1.89–2.87]) and Central regions (OR 1.49 [95% CI 1.19–1.87]), and in those with self-reported histories of heart diseases (OR 1.40 [95% CI 1.07–1.82]), kidney disease (OR 1.80 [95% CI 1.35–2.40]), and digestive diseases (OR 1.54 [95% CI 1.30–1.82]). In contrast, those with an education level of middle school (OR 0.69 [95% CI 0.52–0.90]), or high school or higher (OR 0.60 [95% CI 0.41–0.88]), or who had undertaken some physical activities (OR 0.47, 95% CI 0.29–0.76) were associated with less likelihood of incident symptomatic knee OA.

**Table 3:** Longitudinal analysis of incident symptomatic knee OA with different variables in different models

Variables	Model 1		Model 2	
	OR	95%CI	OR	95%CI
**Gender**				
Male				
Female	**1.97**	**1.65–2.37**	**1.98**	**1.65–2.37**
**Age, years**				
< 50				
50–59	0.88	0.60–1.28	0.88	0.60–1.29
60–69	1.18	0.82–1.71	1.14	0.78–1.64
≥ 70	1.09	0.76–1.57	1.01	0.70–1.47
**Area**				
Urban				
Rural	**1.31**	**1.08–1.59**	**1.32**	**1.08–1.60**
**Region**				
East				
Central	**1.56**	**1.24–1.97**	**1.49**	**1.19–1.87**
West	**2.39**	**1.92–2.99**	**2.33**	**1.89–2.87**
**Education**				
No formal education				
Elementary school	1.14	0.93–1.38	1.08	0.89–1.32
Middle school	**0.70**	**0.54–0.92**	**0.69**	**0.52–0.90**
High school or Vocational school or higher	**0.64**	**0.43–0.93**	**0.60**	**0.41–0.88**
**BMI group (kg/m**^**2**^**)**				
< 18.5				
18.5–24.9	1.08	0.79–1.47	1.10	0.80–1.51
≥ 25.0	1.25	0.89–1.75	1.21	0.87–1.69
**Done some activities** (such as played a sport, social, or other kind of club dancing, doing physical exercise, doing Qigong,et al.)				
No				
Yes	**0.50**	**0.31–0.81**	**0.47**	**0.29–0.76**
**Chronic disease**				
**Hypertension** (Yes vs. No)			1.12	0.92–1.37
**Dyslipidemia** (Yes vs. No)			1.07	0.75–1.52
**Diabetes** (Yes vs. No)			1.30	0.78–2.14
**Chronic lung disease** (Yes vs. No)			1.19	0.92–1.55
**Liver disease** (Yes vs. No)			1.22	0.83–1.80
**Heart disease** (Yes vs. No)			**1.40**	**1.07–1.82**
**Stroke** (Yes vs. No)			1.67	0.97–2.86
**Kidney disease** (Yes vs. No)			**1.80**	**1.35–2.39**
**Digestive disease** (Yes vs. No)			**1.54**	**1.30–1.82**
**Psychiatric disease** (Yes vs. No)			1.07	0.60–1.90
**Asthma** (Yes vs. No)			1.17	0.75–1.81

Note: In model 1, we validated some known risk factors, including gender, age, area, region, education, BMI group, doing physical activities in the last month. In model 2, we explored additional potential risk factors, including histories of hypertension, dyslipidemia, diabetes, chronic lung disease, liver disease, heart disease, stroke, kidney disease, digestive disease, psychiatric disease, and asthma

### Sensitivity analysis

Firstly, we compared the differences of missing data in demographic and clinical variables between in the symptomatic knee OA and non-symptomatic knee OA group and found no significant difference in any variable (Supplementary Table 2). Secondly, during sensitivity analysis, we composed different models to estimate risk factors of incident symptomatic knee OA using complete raw data (i.e. complete case analysis) and multiple imputation data (Supplementary Table 3, models 2 and 3). Furthermore, since the BMI group variable (categorical) had more than 20% of the values missing, while the proportions of missing values in each of the other variables were less than 5%, we added one more model using complete raw data but excluded the BMI group variable (Supplementary Table 3, model 4). The results from the models 3 and 4 were consistent with that those from model 2.

With respect to respondents lost to follow up, we first assessed baseline characteristics between respondents lost to follow-up and respondents included in the final analysis. We found significant differences in several variables (Supplementary Table 4), but in sensitivity analyses, the results from the models 5, 6 and 7 were consistent with that those from model 2. Only two risk factors (i.e. residential area, done physical activities) showed association with increased risk in models 2, 5, and 6, not in model 7 (Supplementary Table 5).

## Discussion

Using data collected from the CHARLS, a national population survey with a 4-year follow-up, our study found that the cumulative incidence of symptomatic knee OA over 4 years among Chinese adults aged ≥45 years was 8.5%. Our study also showed significant variations of the incidence by province. To the best of our knowledge, this is the first study to report the incidence of symptomatic knee OA among the Chinese population. The findings may provide valuable information for health-care policy makers, allowing them to better allocate health-care resources and develop evidence-informed health-care planning by province. In particular, the findings may have important implications for those provinces with higher incidence.

Few studies have examined incident symptomatic knee OA. In the Framingham Osteoarthritis Study, with a ~ 8.1-year follow-up, the incidence rate of symptomatic knee OA was 6.7% (0.8% per year) [12]. In the current study, with a ~ 4-year follow-up, the estimated incidence of symptomatic knee OA is 8.5% (2.1% per year), higher than that in the Framingham Osteoarthritis Study.

Some studies have examined the incidence of radiographic knee OA. One study in the Japan showed that the incidence rate of K/L grade ≥ 2 knee OA was 2.9% per year [10]. Another study in the U.K. showed that the incident rate of K/L grade ≥ 2 knee OA was 2.5% per year [11]. However, a study in the Spain showed that the incidence rate of knee OA was identified using International Classification of Diseases (ICD)-10 codes, was 0.64% per year [13]. However, the definitions of the cases varied among these studies.

The incidence of symptomatic knee OA was significantly higher in women, consistent with previous studies [10, 13, 19, 20]. This is likely due to women predisposed with higher bone mineral density [21]. Consistent with other studies, our analysis found that senior ages were associated with higher risk of symptomatic knee OA [3, 10, 13, 22]. In addition, our study found that the incidence was highest among respondents aged 60–69 years, then decreased after 70 years, possibly because elderly persons generally do less heavy physical activities after the age of 70 years; thus, less heavy physical activities may reduce knee symptoms, which make the incidence of symptomatic knee OA decreased. Our study also found that those with a higher level of education had a lower risk of symptomatic knee OA, consistent with previous studies [9, 23]. This is likely due to those receiving less education being more likely to be employment in physical labor [24]. Overweight/obesity was associated with an increased risk of incident symptomatic knee OA in our study, although it was not significant. Previous studies have shown that overweight/obesity was associated with an increased risk of both radiographic and symptomatic knee OA [15, 22, 25, 26].

In our study, those residing in rural areas or in West part of China had a higher risk of incident symptomatic knee OA. Meanwhile, the provinces with the highest incidence were mainly in the West region. These findings were consistent with previous cross-sectional results [9, 20, 27]. This may be explained by that residents in rural areas often have less-privileged socioeconomic conditions and limited access to health-care resources, while undertaking more physical labor. The difference among the three regions is also attributable to the difference in terrain and socioeconomic imbalance.

We found that people doing certain physical activities (e.g. dancing, body building) often had lower risk of incident symptomatic knee OA, which is similar to the findings of a review about OA [28]. As shown earlier, a light and moderate level of activity may be associated with less subsequent disabilities, such as knee OA [29]. This finding suggested that regular physical activity is always warranted for preventing the development of this condition.

Self-reported hypertension, heart disease, kidney disease, and digestive disease are associated with increased risk of incident symptomatic knee OA, as shown by our study. One set of meta-analyses results showed that hypertension was significantly associated with higher incidence of symptomatic knee OA [30]. One possible explanation is that they share traditional risk factors, such as chronic inflammation. One study confirmed that CVD was a risk factor for knee OA [15]. A higher risk of CVD has also been observed in people with OA [31]. For these analyses, CVD included heart disease. It is possible that heart disease and symptomatic OA have a bidirectional relationship with OA. Similarly, self-reported kidney disease and digestive disease were associated with incidence of symptomatic knee OA is possible that kidney disease and digestive disease may be caused by medication use due to symptomatic knee OA. The chronic inflammation and nonsteroidal anti-inflammatory drug (NSAID) treatment in symptomatic knee OA patients are reported to increase the risk of getting kidney disease and digestive disease [32, 33]. Further studies are warranted to confirm the relationship between these diseases and knee OA. Another possible explanation might be that persons with these diseases have more contact with health care and, thus, are more prone to receive a diagnosis of arthritis.

Our study has several strengths. Firstly, the CHARLS included a nationwide representative sample of middle-aged and older adults. The findings are generalizable to the Chinese population. Secondly, the survey was conducted using a strict quality-control program, and the study participants were chosen according to a strict multistage probability sampling procedure. Finally, we reported both the incidence and associated risk factors for symptomatic knee OA, which is helpful for healthcare policy development and clinical practice.

Our study also has limitations. Firstly, the respondents in the CHARLS did not undergo radiographic assessment, and hence the diagnosis of symptomatic knee OA was based on self-reported knee pain and self-reported arthritis diagnosis by a physician, which differed from the diagnostic criteria used in other studies [34]. However, this definition provided the best available diagnosis for symptomatic knee OA in CHARLS and has been used in several published studies [9, 35]. Secondly, data for other chronic diseases were also collected on the basis of self-reporting. Hence, the associations, which we observed between chronic diseases and symptomatic knee OA, might be confounded by the increased contact with health care professionals in patients with long-term disease, which in turn leads to increased reception of a diagnosis of arthritis. However, our findings were generally consistent with previous studies [15, 30]. Thirdly, the question about OA diagnosis did not distinguish between OA and other arthritis (e.g. rheumatoid arthritis, gout et al.), which would lead to overestimating the incidence of OA. Nevertheless, in economically backward regions, the residents may less likely to visit physicians, which may result in underestimating the incidence of OA. Fourthly, there is 4 years of longitudinal data in our study, the results would be more reliable for a longer cohort. Finally, findings regarding risk factors should be interpreted with caution since different definition of knee OA have different risk factors, and our findings may differ from other studies focusing on other diagnosis of knee OA.

## Conclusions

Using data from the CHARLS, we observed that the cumulative incidence of symptomatic knee OA among middle-aged or older Chinese adults was high, was even more common among females, and varied by province and region. Those adults not undertaking physical activities, or presenting with heart disease, kidney disease, or digestive diseases had a higher likelihood of developing incident symptomatic knee OA. Further research with more reliable diagnosis need to confirm our findings. The findings may be intriguing for health care, and may have important implications for practitioners and policy makers, particularly those from developing countries.

## Supplementary information

**Additional file 1 Table S1.** The questions used to assess symptomatic knee OA and other health conditions

**Additional file 2 Table S2.** Missing data in baseline variables in the symptomatic knee OA and non-symptomatic knee OA groups

**Additional file 3 Table S3.** Sensitivity analysis for estimating risk factors of symptomatic knee OA using complete case analysis (Model 2), multiple imputed data (Model 3), and complete case analysis without BMI (Model 4)

**Additional file 4 Table S4.** Baseline characteristics in respondents lost to follow-up and respondents included in final analysis

**Additional file 5 Table S5.** Sensitivity analysis for estimate risk factors of symptomatic knee OA using respondents lost to follow up in different models

## Data Availability

All data collected in the CHARLS are maintained at the National School of Development of Peking University, Beijing, China. The datasets are available from http://charls.pku.edu.cn/pages/data/111/zh-cn.html.

## Abbreviations

OA: Osteoarthritis
CHARLS: China Health and Retirement Longitudinal Study
PPS: Probability-proportional-to-size
GDP: Gross domestic product
PSUs: Primary sampling units
BMI: Body mass index
WHO: World Health Organization
