# Supplementary material for: Incidence and risk factors of symptomatic knee osteoarthritis among the Chinese population: analysis from a nationwide longitudinal study
Source: BMC Public Health. 2020 Oct 1;20:1491. doi: 10.1186/s12889-020-09611-7 (PMC7528331; doi:10.1186/s12889-020-09611-7)
Supplement: Supplementary file 1 — Additional file 1 Table S1. The questions used to assess symptomatic knee OA and other health conditions [file 12889_2020_9611_MOESM1_ESM.docx]

Supplementary table 1. The questions used to assess symptomatic knee OA and other health conditions

| **Number** | **Questions** | **Answers** |
| --- | --- | --- |
| 1 | Have you been diagnosed with [conditions listed below, read one by one] by a doctor?  [IWER: Read one by one. 1=yes, 2=no.] | (1) Hypertension  (2) Dyslipidemia (elevation of low density lipoprotein, triglycerides (TGs), and total cholesterol, or a low high density lipoprotein level)  (3) Diabetes or high blood sugar  (4) Cancer or malignant tumor (excluding minor skin cancers)  (5) Chronic lung diseases, such as chronic bronchitis, emphysema (excluding tumors, or cancer)  (6) Liver disease (except fatty liver, tumors, and cancer)  (7) Heart attack, coronary heart disease, angina, congestive heart failure, or other heart problems  (8) Stroke  (9) Kidney disease (except for tumor or cancer)  (10) Stomach or other digestive disease (except for tumor or cancer)  (11) Emotional, nervous, or psychiatric problems  (12) Memory-related disease  (13) Arthritis or rheumatism  (14) Asthma |
| 2 | Are you often troubled with any body pains? | (1) Yes (2) No |
| 3 | On what part of your body do you feel pain? Please list all parts of body you are currently feeling pain. | (1) Head (Headache) (2) Shoulder (3) Arm (4) Wrist (5) Fingers  (6) Chest (7) Stomach (Stomachache) (8) Back (9) Waist  (10) Buttocks (11) Leg (12) Knees (13) Ankle (14) Toes  (15) Neck (16) Other |
