# Supplementary material for: Incidence and risk factors of symptomatic knee osteoarthritis among the Chinese population: analysis from a nationwide longitudinal study
Source: BMC Public Health. 2020 Oct 1;20:1491. doi: 10.1186/s12889-020-09611-7 (PMC7528331; doi:10.1186/s12889-020-09611-7)
Supplement: Supplementary file 2 — Additional file 2 Table S2. Missing data in baseline variables in the symptomatic knee OA and non-symptomatic knee OA groups [file 12889_2020_9611_MOESM2_ESM.docx]

Supplementary table 2. Missing data in baseline variables in the symptomatic knee OA and non-symptomatic knee OA groups

| **Variables** | Non-symptomatic knee OA(n=11881)  n (%) | | Symptomatic knee OA(n=1196)  n (%) | P-value |
| --- | --- | --- | --- | --- |
| **Gender** |  |  | | **0.5257** |
| Missing | 4(0.03) | 0(0) | |  |
| None missing | 11877(99.97) | 1196(100) | |  |
| **BMI group (kg/m^2^)** |  |  | | **0.1047** |
| Missing | 2348(19.76) | 213(17.81) | |  |
| None missing | 9533(80.24) | 983(82.19) | |  |
| **Hypertension** |  |  | | **0.2408** |
| Missing | 59 ( 0.50) | 9 ( 0.75) | |  |
| None missing | 11822 ( 99.50) | 1187 ( 99.25) | |  |
| **Dyslipidemia** |  |  | | **0.0166** |
| Missing | 211(1.78) | 33(2.76) | |  |
| None missing | 11670(98.22) | 1163(97.24) | |  |
| **Diabetes** |  |  | | **0.1554** |
| Missing | 101 ( 0.85) | 15 (1.25) | |  |
| None missing | 11780 ( 99.15) | 1181 ( 98.75) | |  |
| **Chronic lung** |  |  | | **0.6845** |
| Missing | 41(0.35) | 5(0.42) | |  |
| None missing | 11840(99.65) | 1191(99.58) | |  |
| **Liver disease** |  |  | | **0.6169** |
| Missing | 75(0.63) | 9(0.75) | |  |
| None missing | 11806(99.37) | 1187(99.25) | |  |
| **Heart disease** |  |  | | **0.0870** |
| Missing | 63(0.53) | 11(0.92) | |  |
| None missing | 11818(99.47) | 1185(99.08) | |  |
| **Stroke** |  |  | | **0.2866** |
| Missing | 28(0.24) | 1(0.08) | |  |
| None missing | 11853(99.76) | 1195(99.92) | |  |
| **Kidney disease** |  |  | | **0.3221** |
| Missing | 63(0.53) | 9(0.75) | |  |
| None missing | 11818(99.47) | 1187(99.25) | |  |
| **Digestive disease** |  |  | | **0.0592** |
| Missing | 26(0.22) | 6(0.5) | |  |
| None missing | 11855(99.78) | 1190(99.5) | |  |
| **Psychiatric disease** |  |  | | **0.2556** |
| Missing | 44(0.37) | 7(0.59) | |  |
| None missing | 11837(99.63) | 1189(99.41) | |  |
| **Asthma** |  |  | | **0.8797** |
| Missing | 43(0.36) | 4(0.33) | |  |
| None missing | 11838(99.64) | 1192(99.67) | |  |

Note: There was no missing value in the following four variables: Age, Area, Region, and Done some activities.
