# Supplementary material for: Incidence and risk factors of symptomatic knee osteoarthritis among the Chinese population: analysis from a nationwide longitudinal study
Source: BMC Public Health. 2020 Oct 1;20:1491. doi: 10.1186/s12889-020-09611-7 (PMC7528331; doi:10.1186/s12889-020-09611-7)
Supplement: Supplementary file 3 — Additional file 3 Table S3. Sensitivity analysis for estimating risk factors of symptomatic knee OA using complete case analysis (Model 2), multiple imputed data (Model 3), and complete case analysis without BMI (Model 4) [file 12889_2020_9611_MOESM3_ESM.docx]

Supplementary table 3. Sensitivity analysis for estimating risk factors of symptomatic knee OA using complete case analysis (Model 2), multiple imputed data (Model 3), and complete case analysis without BMI (Model 4)

| **Variables** | Model 2 | Model 3 | Model 4 |
| --- | --- | --- | --- |
| **Gender** |  |  |  |
| Male |  |  |  |
| Female | **1.98(1.65-2.37)** | **2.03(1.73-2.38)** | **2.12(1.79-2.50)** |
| **Age, years** |  |  |  |
| <50 |  |  |  |
| 50-59 | 0.88(0.60-1.29) | 1.08(0.79-1.47) | 1.11(0.81-1.54) |
| 60-69 | 1.14(0.79-1.65) | 1.29(0.95-1.75) | 1.31(0.96-1.79) |
| ≥70 | 1.01(0.70-1.47) | 1.11(0.81-1.52) | 1.12(0.82-1.54) |
| **Area** |  |  |  |
| Urban |  |  |  |
| Rural | **1.31(1.08-1.60)** | **1.47(1.24-1.74)** | **1.44(1.21-1.71)** |
| **Region** |  |  |  |
| East |  |  |  |
| Central | **1.49(1.19-1.87)** | **1.55(1.27-1.90)** | **1.55(1.26-1.90)** |
| West | **2.33(1.88-2.87)** | **2.53(2.10-3.05)** | **2.45(2.03-2.97)** |
| **Education** |  |  |  |
| No formal education |  |  |  |
| Elementary school | 1.08(0.88-1.31) | 1.04(0.87-1.24) | 1.05(0.87-1.25) |
| Middle school | **0.68(0.52-0.90)** | **0.63(0.49-0.80)** | **0.65(0.51-0.84)** |
| High school or Vocational school or higher | **0.60(0.41-0.88)** | **0.54(0.39-0.74)** | **0.54(0.39-0.76)** |
| **BMI group (kg/m^2^)** |  |  |  |
| <18.5 |  |  |  |
| 18.5-24.9 | 1.10(0.80-1.51) | 1.18(0.88-1.58) |  |
| ≥25.0 | 1.21(0.87-1.69) | 1.22(0.90-1.67) |  |
| **Done some activities**(such as played a sport, social, or other kind of club dancing, doing physical exercise, doing Qigong,et al. ) | | | |
| No |  |  |  |
| Yes | **0.47(0.29-0.76)** | **0.54(0.34-0.86)** | **0.54(0.34-0.87)** |
| **Chronic disease** |  |  |  |
| **Hypertension** (Yes vs. No) | 1.12(0.92-1.36) | 1.14(0.96-1.37) | 1.20(1.01-1.44) |
| **Dyslipidemia** (Yes vs. No) | 1.06(0.74-1.51) | 1.03(0.76-1.40) | 1.05(0.77-1.44) |
| **Diabetes** (Yes vs. No) | 1.30(0.78-2.14) | 1.39(0.90-2.15) | 1.37(0.87-2.14) |
| **Chronic lung disease** (Yes vs. No) | 1.20(0.93-1.55) | 1.28(1.01-1.61) | 1.21(0.95-1.54) |
| **Liver disease** (Yes vs. No) | 1.21(0.82-1.79) | 1.26(0.90-1.77) | 1.18(0.83-1.67) |
| **Heart disease** (Yes vs. No) | **1.39(1.06-1.82)** | **1.51(1.20-1.91)** | **1.53(1.20-1.94)** |
| **Stroke** (Yes vs. No) | 1.67(0.98-2.86) | 1.34(0.83-2.17) | 1.33(0.80-2.19) |
| **Kidney disease** (Yes vs. No) | **1.80(1.35-2.40)** | **1.62(1.25-2.10)** | **1.75(1.34-2.28)** |
| **Digestive disease** (Yes vs. No) | **1.53(1.29-1.81)** | **1.58(1.36-1.84)** | **1.55(1.33-1.81)** |
| **Psychiatric disease** (Yes vs. No) | 1.04(0.58-1.85) | 0.82(0.50-1.36) | 0.74(0.43-1.25) |
| **Asthma** (Yes vs. No) | 1.15(0.75-1.79) | 1.30(0.90-1.88) | 1.33(0.91-1.94) |

Note: Model 2 used a complete case analysis with all variables (BMI group variable included). Model 3 used a multiple imputation analysis with all variables. Model 4 used a complete case analysis and excluded the BMI group variable.
