# Supplementary material for: Incidence and risk factors of symptomatic knee osteoarthritis among the Chinese population: analysis from a nationwide longitudinal study
Source: BMC Public Health. 2020 Oct 1;20:1491. doi: 10.1186/s12889-020-09611-7 (PMC7528331; doi:10.1186/s12889-020-09611-7)
Supplement: Supplementary file 4 — Additional file 4 Table S4. Baseline characteristics in respondents lost to follow-up and respondents included in final analysis [file 12889_2020_9611_MOESM4_ESM.docx]

Supplementary table 4. Baseline characteristics in respondents lost to follow-up and respondents included in final analysis

| **Variables** | Respondents lost to follow-up  (n=2833)  n (%) | | Respondents included in final analysis  (n=13077)  n (%) | P-value |
| --- | --- | --- | --- | --- |
| **Gender** |  |  | | 0.0112 |
| Male | 767(52.04) | 6347(48.55) | |  |
| Female | 707(47.96) | 6726(51.45) | |  |
| **Age, years** |  |  | | <0.0001 |
| <50 | 245(8.65) | 908(6.94) | |  |
| 50-59 | 793(27.99) | 4043(30.92) | |  |
| 60-69 | 776(27.39) | 4776(36.52) | |  |
| ≥70 | 1019(35.97) | 3350(25.62) | |  |
| **Area** |  |  | | <0.0001 |
| Urban | 1709(60.32) | 4937(37.75) | |  |
| Rural | 1124(39.68) | 8140(62.25) | |  |
| **Region** |  |  | | 0.5282 |
| East | 1006(35.51) | 4760(36.4) | |  |
| Central | 947(33.43) | 4237(32.4) | |  |
| West | 880(31.06) | 4080(31.2) | |  |
| **Education** |  |  | | <0.0001 |
| No formal education | 706(24.99) | 3441(26.31) | |  |
| Elementary school | 971(34.37) | 5230(39.99) | |  |
| Middle school | 574(20.32) | 2839(21.71) | |  |
| High school or Vocational school or higher | 574(20.32) | 1567(11.98) | |  |
| **BMI group (kg/m^2^)** |  |  | | <0.0001 |
| <18.5 | 173(9.98) | 678(6.45) | |  |
| 18.5-24.9 | 1073(61.88) | 6615(62.9) | |  |
| ≥25.0 | 488(28.14) | 3223(30.65) | |  |
| **Done some activities** (such as playeda sport, social, or other kind of club dancing, doing physical exercise, doing Qigong,et al. ) | | | | <0.0001 |
| Yes | 284(10.02) | 705(5.39) | |  |
| No | 2549(89.98) | 12372(94.61) | |  |
| **Hypertension** |  |  | | <0.0001 |
| Yes | 817(28.99) | 2930(22.52) | |  |
| No | 2001(71.01) | 10079(77.48) | |  |
| **Dyslipidemia** |  |  | | 0.0281 |
| Yes | 281(10.12) | 1129(8.8) | |  |
| No | 2497(89.88) | 11704(91.2) | |  |
| **Diabetes** |  |  | | <0.0001 |
| Yes | 200(7.12) | 670(5.17) | |  |
| No | 2609(92.88) | 12291(94.83) | |  |
| **Chronic lung** |  |  | | <0.0001 |
| Yes | 315(11.16) | 1130(8.67) | |  |
| No | 2508(88.84) | 11901(91.33) | |  |
| **Liver disease** |  |  | | 0.5766 |
| Yes | 100(3.55) | 434(3.34) | |  |
| No | 2717(96.45) | 12559(96.66) | |  |
| **Heart disease** |  |  | | <0.0001 |
| Yes | 393(13.92) | 1335(10.27) | |  |
| No | 2430(86.08) | 11668(89.73) | |  |
| **Stroke** |  |  | | <0.0001 |
| Yes | 117(4.14) | 237(1.82) | |  |
| No | 2712(95.86) | 12811(98.18) | |  |
| **Kidney disease** |  |  | | 0.0865 |
| Yes | 168(5.96) | 671(5.16) | |  |
| No | 2652(94.04) | 12334(94.84) | |  |
| **Digestive disease** |  |  | | <0.0001 |
| Yes | 486(17.2) | 2671(20.48) | |  |
| No | 2339(82.8) | 10374(79.52) | |  |
| **Psychiatric disease** |  |  | | 0.3675 |
| Yes | 42(1.49) | 166(1.27) | |  |
| No | 2782(98.51) | 12860(98.73) | |  |
| **Asthma** |  |  | | 0.0006 |
| Yes | 121(4.28) | 394(3.02) | |  |
| No | 2705(95.72) | 12636(96.98) | |  |
