# Supplementary material for: Incidence and risk factors of symptomatic knee osteoarthritis among the Chinese population: analysis from a nationwide longitudinal study
Source: BMC Public Health. 2020 Oct 1;20:1491. doi: 10.1186/s12889-020-09611-7 (PMC7528331; doi:10.1186/s12889-020-09611-7)
Supplement: Supplementary file 5 — Additional file 5 Table S5. Sensitivity analysis for estimate risk factors of symptomatic knee OA using respondents lost to follow up in different models [file 12889_2020_9611_MOESM5_ESM.docx]

Supplementary table 5. Sensitivity analysis for estimate risk factors of symptomatic knee OA using respondents lost to follow up in different models

| **Variables** | Model 2 | Model 5 | | Model6 | Model7 |  |
| --- | --- | --- | --- | --- | --- | --- |
| **Gender** |  | |  |  |  | |
| Male |  | |  |  |  | |
| Female | **1.98(1.65-2.37)** | | **1.91(1.64-2.21)** | **1.99(1.66-2.38)** | **1.29(1.10-1.51)** | |
| **Age, years** |  | |  |  |  | |
| <50 |  | |  |  |  | |
| 50-59 | 0.88(0.60-1.29) | | 1.01(0.76-1.35) | 0.89(0.61-1.31) | 0.92(0.65-1.29) | |
| 60-69 | 1.14(0.79-1.65) | | 1.18(0.90-1.56) | 1.17(0.81-1.69) | 0.95(0.69-1.30) | |
| ≥70 | 1.01(0.70-1.47) | | 1.01(0.76-1.33) | 0.98(0.68-1.43) | 1.15(0.85-1.57) | |
| **Area** |  | |  |  |  | |
| Urban |  | |  |  |  | |
| Rural | **1.31(1.08-1.60)** | | **1.42(1.22-1.65)** | **1.40(1.15-1.70)** | **0.77(0.65-0.91)** | |
| **Region** |  | |  |  |  | |
| East |  | |  |  |  | |
| Central | **1.49(1.19-1.87)** | | **1.61(1.34-1.94)** | **1.48(1.18-1.85)** | 1.10(0.91-1.34) | |
| West | **2.33(1.88-2.87)** | | **2.69(2.25-3.22)** | **2.35(1.90-2.90)** | **1.28(1.06-1.55)** | |
| **Education** |  | |  |  |  | |
| No formal education |  | |  |  |  | |
| Elementary school | 1.08(0.88-1.31) | | 0.99(0.84-1.17) | 1.09(0.90-1.33) | 0.96(0.80-1.15) | |
| Middle school | **0.68(0.52-0.90)** | | **0.59(0.47-0.74)** | **0.70(0.53-0.92)** | **0.77(0.61-0.97)** | |
| High school or Vocational school or higher | **0.60(0.41-0.88)** | | **0.55(0.41-0.73)** | **0.58(0.40-0.85)** | 1.00(0.74-1.36) | |
| **BMI group (kg/m^2^)** |  | |  |  |  | |
| <18.5 |  | |  |  |  | |
| 18.5-24.9 | 1.10(0.80-1.51) | | 1.12(0.87-1.45) | 1.14(0.84-1.57) | 0.81(0.64-1.02) | |
| ≥25.0 | 1.21(0.87-1.69) | | 1.12(0.85-1.48) | 1.25(0.90-1.75) | 0.83(0.64-1.08) | |
| **Done some activities**(such as played a sport, social, or other kind of club dancing, doing physical exercise, doing Qigong,et al. ) | | | | | | |
| No |  | |  |  |  | |
| Yes | **0.47(0.29-0.76)** | | **0.55(0.38-0.80)** | **0.45(0.28-0.73)** | 1.06(0.76-1.46) | |
| **Chronic disease** |  | |  |  |  | |
| **Hypertension** (Yes vs. No) | 1.12(0.92-1.36) | | 1.16(0.99-1.37) | 1.10(0.91-1.34) | 1.13(0.95-1.35) | |
| **Dyslipidemia** (Yes vs. No) | 1.06(0.74-1.51) | | 0.96(0.73-1.26) | 1.08(0.76-1.55) | 0.86(0.67-1.11) | |
| **Diabetes** (Yes vs. No) | 1.30(0.78-2.14) | | 1.23(0.84-1.80) | 1.27(0.77-2.10) | 1.34(0.84-2.15) | |
| **Chronic lung disease** (Yes vs. No) | 1.20(0.93-1.55) | | **1.32(1.08-1.60)** | 1.15(0.89-1.49) | 1.12(0.91-1.37) | |
| **Liver disease** (Yes vs. No) | 1.21(0.82-1.79) | | 1.02(0.75-1.40) | 1.20(0.82-1.76) | 1.10(0.82-1.48) | |
| **Heart disease** (Yes vs. No) | **1.39(1.06-1.82)** | | **1.57(1.27-1.94)** | **1.41(1.08-1.85)** | 1.18(0.96-1.44) | |
| **Stroke** (Yes vs. No) | 1.67(0.98-2.86) | | 1.30(0.86-1.96) | 1.60(0.94-2.72) | 1.44(0.97-2.13) | |
| **Kidney disease** (Yes vs. No) | **1.80(1.35-2.40)** | | **1.76(1.38-2.25)** | **1.74(1.32-2.31)** | **1.34(1.05-1.72)** | |
| **Digestive disease** (Yes vs. No) | **1.53(1.29-1.81)** | | **1.61(1.40-1.85)** | **1.54(1.30-1.82)** | **1.24(1.03-1.48)** | |
| **Psychiatric disease** (Yes vs. No) | 1.04(0.58-1.85) | | 0.71(0.43-1.18) | 1.14(0.65-2.02) | 0.89(0.55-1.43) | |
| **Asthma** (Yes vs. No) | 1.15(0.75-1.79) | | 1.23(0.89-1.72) | 1.19(0.77-1.82) | 1.18(0.87-1.61) | |

Note: Model 2 used a complete case analysis (Respondents lost to follow up not included). Model 5 used a multiple imputation analysis for respondents lost to follow up. Model 6 imputed the none symptomatic knee OA for respondents lost to follow up. Model 7 imputed the symptomatic knee OA for respondents lost to follow up.
